# Determinants of frailty in primary care patients with COPD: the Greek UNLOCK study

**DOI:** 10.1186/s12890-019-0824-8

**Published:** 2019-03-15

**Authors:** Despo Ierodiakonou, Maria Kampouraki, Ioannis Poulonirakis, Polyvios Papadokostakis, Eleftheria Lintovoi, Dimitris Karanassos, Kyriakos Maltezis, Maria Chorti, Evangelos Petrovitsos, Sofia Dimopoulou, Sam Hamind, Ioannis Gialamas, Polyxeni Athanasiou, Vasiliki Bempi, Irene Lambraki, Ioanna Tsiligianni

**Affiliations:** 1 Department of Social Medicine, Faculty of Medicine, University of Crete, Voutes Campus, GR-71003 Heraklion, Crete Greece; 2 Heraklion University Hospital, Heraklion, Crete Greece; 3 Primary care practice, Health Center of Moires, Heraklion, Crete Greece; 4 Primary care practice, Health Center of Agia Varvara, Heraklion, Crete Greece; 5 Primary care practice, Garipa, Herkalion, Crete Greece; 6 Primary care practice, Corfu, Greece; 7 Primary care practice, Akraifnio, Boeotia, Greece; 8 Primary care practice, Attica, Greece; 9 Primary care practice, Chalkidiki, Greece; 10 Primary care practice, Santorini, Cyclades Islands, Greece; 11 Primary care practice, Health Center of Sitia, Sitia General Hospital, Lasithi, Crete Greece

**Keywords:** COPD, Fraitly, Severity, Symptoms, Exacerbations, CAT, mMRC

## Abstract

**Background:**

Frailty is a state of increased vulnerability that has a significant risk of unfavorable outcomes such as increased dependency and/or death, but little is known about frailty in people with chronic obstructive pulmonary disease (COPD).

**Method:**

We aimed to determine the prevalence of frailty in COPD patients and to identify the associated risk factors. Two hundred fifty-seven COPD patients enrolled from primary care in Greece between 2015 and 2016. Physicians used structured interviews to collect cross-sectional data including demographics, medical history, symptoms and COPD Assessment Tool (CAT) or modified Medical Research Council Dyspnea scale (mMRC) score. Patients were classified into severity groups according to GOLD 2017 guidelines. Participants completed the The Frail Non-Disabled (FiND) questionnaire, exploring the frailty and disability domains. In the present analyses, frail patients with and without mobility disability were pooled and were compared to non-frail patients. Factors associated with frailty were analyzed using univariate and multivariate logistic regression.

**Results:**

Mean (SD) age was 65 (12.3) with 79% males. The majority of patients suffered with frailty (82%) of which 76.8% had mobility disability. 84.2% were married/with partner and 55.4% retired. 55.6% were current smokers. Uncontrolled disease (≥10 CAT score) was reported in 91.1% and 37.2% of patients had ≥2 exacerbations in the past year. Dyspnea (38%) and cough (53.4%) were the main symptoms. Main comorbidities were hypertension (72.9%), hyperlipidaemia (24.6%) and diabetes (11%).

Risk of frailty was significantly increased with age (OR; 95%CI: 1.05; 1.02–1.08), hypertension (2.25; 1.14–4.45), uncontrolled disease (≥10 CAT score 4.65; 1.86–11.63, ≥2 mMRC score 5.75 (2.79–11.85) or ≥ 2 exacerbations 1.73; 1.07–2.78), smoking cessation (ex compared to current smokers: 2.37; 1.10–5.28) and GOLD status (B&D compared to A&C groups: CAT-based 4.65; 1.86–11.63; mMRC-based: 5.75; 2.79–11.85). In multivariate regression smoking cessation and GOLD status remained significant. Gender, body mass index, occupational or marital status, symptoms and other comorbidities were not significant.

**Conclusions:**

Frailty with mobility disability is common in COPD patients and severity of disease increases the risk. It is possible that frail patients are more likely to quit smoking perhaps because of their disability and uncontolled disease. Routine assessment of frailty in addition to COPD control may allow early interventions for preventing or delaying progression of frailty and improvement in COPD disease.

## Background

Frailty is a known health problem of the senior population and of subjects suffering from chronic diseases that enhances the risk of adverse outcomes, including accidental falls, admissions to hospitals, institutionalization, and mortality [1–3]. The Frailty Consensus Conference defined physical frailty as “a medical syndrome with multiple causes and contributors that is characterized by diminished strength, endurance, and reduced physiologic function that increases an individual’s vulnerability for developing increased dependency and/or death” [4]. Identifying frail older subjects living in the community has recently been well accepted as the cornerstone for preventing frailty’s adverse health outcomes [5].

Even though the frequency of frailty may vary depending on the assessment method used, overall it is estimated to affect 10.7% of persons aged ≥65 [6], and prevalence can be as high as 50% or greater depending on the frailty definition used [7, 8]. Prevalence increases with age, female gender and with a higher number of comorbidities [6, 9]. The shared underlying pathways linking frailty to aging and other chronic diseases remain unclear, but some of the proposed biological mechanisms include chronic systemic inflammation, musculoskeletal and neuroendocrine dysfunction [10, 11].

Chronic obstructive pulmonary disease (COPD) is a condition commonly resulting in vulnerable elderly populations. COPD is closely related to frailty with shared risk factors such as aging and smoking and common mechanisms of dysregulated inflammation and endocrine dysfunction [12]. Although primarily a lung disease, COPD is recognized to have extra-pulmonary effects, including musculoskeletal dysfunction, nutritional imbalance with weight loss, and cardiovascular events, among others [13, 14]. Studies exploring the issue of frailty in COPD are fewer and those that exist, suggest that the prevalence of frailty is higher in COPD patients as compared to the general population; but the prevalence given in the different studies varies depending on the instrument used for screening and the population [8, 9, 15]. Galizia et al. reported that frailty increased the mortality risk by 80% in patients suffering from COPD, while Lahousse et al. suggested that in addition to COPD severity and comorbidities, frailty predicts risk of mortality [9, 16]. Moreover frailty has been suggested as a risk factor of readmissions within 90 days of hospitalization for acute COPD exacerbations [17].

Multiple tools exist for screening of frailty in clinical settings but most of them are not designed to be self-administered [18, 19]. If the screening has to target large populations, this may become an issue especially for long-term follow up in primary care. The “Frail non-Disabled” (FiND) questionnaire is constructed based on the widely used frailty phenotype (including 5 main criteria: unintentional weight loss (4.5 kg in past year), self-reported exhaustion, weakness (grip strength), slow walking speed, and low physical activity) [3], but it also includes a specific section for exploring the presence of mobility disability (an early stage of the disabling process). In a study the FiND questionnaire presented a good precision in identifying community dwelling frail older persons without mobility disability [20].

In the current cross-sectional study we used the FiND questionnaire in primary care settings and we aimed to determine the prevalence of frailty (with and without mobility disability)within COPD patients, and to identify population and disease characteristics that may increase the risk of frailty in COPD patients.

## Methods

This study consists of the Greek national branch of the UNLOCK (Uncovering and Noting Long-term Outcomes in COPD and asthma to enhance Knowledge), an international collaboration between primary care researchers to coordinate and share datasets of relevant diagnostic and follow-up variables for COPD and asthma management in primary care. It was set up by members of International Primary Care Respiratory Group and the first author IT was responsible for developing the Greek database. The protocol summary was published in 2010 [21]. The study was approved by the local medical ethics committee of the University Hospital of Crete Greece (protocol number 7985), and the patients gave written informed consent.

A sample of 257 COPD patients was enrolled between 2015 and 2016 from 53 primary care facilities in Greece. A convenient sampling method was used to select COPD patients living in rural and semi-urban areas served by primary care facilities across Greece. The participation rate among areas varied from 78 to 91%. General practitioners with structured interviews collected cross-sectional information including demographic characteristics, medical history, lifestyle, respiratory symptoms, annual number of exacerbations and hospitalisations due to respiratory illness, and medication used for COPD management. The COPD Assessment Test (CAT) [22] and Modified Medical Research Council (mMRC) Dyspnea Scale [23] were completed and scores were calculated. COPD patients were classified according to Global Initiative for Chronic Obstructive Lung Disease (GOLD) 2017 guidelines e.g., ABCD grading system [24]. The ABCD grading system considers COPD health status, assessed by CAT or mMRC, along with exacerbation frequency and need for hospitalisation (A is better, D is worse). We classified COPD patients to A-D groups based on both CAT and mMRC tools.

For screening for frailty, participants completed the FiND questionnaire [20], including frailty and disability domains. The FiND is a tool designed to be applied in the community and primary care, can be self-administered, be completed in approximately 1 min, and it does not require record data or clinical assessment [25]. We also selected the FiND questionnaire as a tool for measuring frailty because we aimed to identify COPD subjects at an early stage of the syndrome (e.g. without mobility disability). The FiND questionnaire consists of five different questions/items (Table 1). Two questions are specifically aimed at identifying individuals with mobility disability (items A and B). Three additional questions assess conditions commonly considered as components of the frailty syndrome [3] (items C: weight loss, D: exhaustion and E: sedentary behaviour). If A + B ≥ 1, the individual is considered as “disabled”. If A + B = 0 and C + D + E ≥ 1, the individual is considered as “frail”. Patients positive for disability could also be positive for frailty. If A + B + C + D + E = 0, were considered as “robust” (without frailty or disability) at the FiND questionnaire. In the present analyses, disabled and frail (without mobility disability) patients were pooled and were compared to robust patients.

**Table 1:** The “Frail non-Disabled” (FiND) questionnaire

Domain	Questions	Answers	Score
*Disability*	A. Have you any difficulties at walking 400 m?	a. No or some difficulties	0
		b. A lot of difficulties or unable	1
	B. Have you any difficulties at climbing up a flight of stairs?	a. No or some difficulties	0
		b. A lot of difficulties or unable	1
*Frailty*	C. During the last year, have you involuntarily lost more than 4.5 kg?	a. No	0
		b. Yes	1
	D. How often in the last week did you feel than everything you did was an effort or that you could not get going?	a. Rarely or sometime (twice or less/week)	0
		b. Often or almost always (3 or more times per week)	1
	E. Which is your level of physical activity?	a. Regular physical activity (at least 2–4 h per week)	0
		b. None or mainly sedentary	1

Items A and B define disability domains and items C, D and E define frailty domains. If A + B ≥ 1, the individual is considered as “disabled”. If A + B = 0 and C + D + E ≥ 1, the individual is considered as “frail”. Participants reporting no mobility disability as well as no frailty criterion e.g. If A + B + C + D + E = 0, are considered as “robust” (non-frail). Subjects positive for disability could also have positive frailty domains

Table adopted by Cesari et al. [20]

### Statistical analysis

Descriptive statistics were presented as a percentage for categorical variables and as mean (standard deviation) for normally distributed variables or as median (minimum-maximum) for continuous variables without a normal distribution. Correlation between CAT and mMRC scores were tested with Pearson Rho coefficient. Chi-square test and T-tests or Mann-Witney tests were used to identify differences between frail and robust COPD patients for qualitative or quantitative variables respectively. A *P*-value of < 0.05 was considered a statistically significant difference. Effects of the factors associated with frailty were evaluated using univariate and multivariate logistic regression analyses. In univariate analysis, crude odds ratio (OR) and 95% confidence intervals (95%CI) was used to examine the strength of association of the factors examined with frailty. Factors with a *P*-value of <0.05 were then entered into a multivariate logistic regression model and adjusted OR (95%CI) was used to determine whether significant factors are independent from each other. Data management and statistical analyses were performed in IBM-SPSS Statistics software (version 23).

## Results

The main population characteristics are presented in Table 2. Mean (SD) population age was 65 (12.3) with 204 (79.4%) males. The majority were married/with partner (208 (84.2%)) and retired (123 (55.4%)). One hundred and forty three (55.6%) were current smokers. CAT score was positively correlated with mMRC score (rho = 0.55; *p*-value< 0.001). With the use of CAT and mMRC tools, uncontrolled/poor health status was reported in 224 (91.1%) and 154 (60.6%), respectively. Seventy seven (37.2%) of the patients had at least 2 exacerbations in the last 12 months. Based on GOLD 2017 guidelines, group B was the largest followed by groups D, A and C. The majority of patients reported dyspnea (135 (53.4%)) as their main symptom, followed by cough (96 (37.9%)). Most subjects suffered from cardiometabolic diseases such as hypertension (186 (72.9%)), hyperlipidemia (63 (24.6%)) and type II diabetes mellitus (28(11%)).

**Table 2:** Population characteristics

	TOTAL	NON-FRAIL	FRAIL-DISABLE	*P*-VALUE^a^
	*N* = 257	*N* = 45 (18%)	*N* = 208 (82%)^b^	
Age; mean (SD)	65 (12.3)	57.3 (11.3)	64.7 (11.6)	**<0.001**
BMI; mean (SD)	29 (5.3)	29 (4.7)	29 (5.3)	0.849
Males; n (%)	204 (79.4)	36 (80.0)	162 (79.0)	0.884
Marital status; n (%)				**0.005**
Married/with partner	208 (84.2)	35 (81.4)	171 (85.1)	
Widower	24 (9.7)	1 (2.3)	22 (10.9)	
Divorced or never married	15 (6.1)	7 (16.3)	8 (4.0)	
Occupational status; n (%)				0.088
Employed	74 (33.3)	17 (45.9)	53 (29.4)	
Unemployed/housewife	25 (11.3)	5 (13.5)	20 (11.1)	
Retired	123 (55.4)	15 (40.5)	107 (59.4)	
Smoking status; n (%)				0.091
Current	143 (55.6)	31 (68.9)	106 (51.7)	
Ex	83 (32.3)	9 (20.0)	73 (35.6)	
Never	31 (12.1)	5 (11.1)	26 (12.7)	
Pack-years; median (min-max)	40 (10–200)	40 (20–100)	40 (10–200)	0.197
Main symptom; n (%)				0.458
Cough	96 (37.9)	15 (34.1)	76 (37.4)	
Dyspnea	135 (53.4)	23 (17.2)	111 (54.7)	
Else (Wheezing, chest tightness, phlegm)	22 (8.7)	6 (13.6)	16 (7.9)	
CAT symptom severity; n (%)				**0.002**
< 10	22 (8.9)	10 (23.3)	12 (6.1)	
≥10	224 (91.1)	33 (77.7)	184 (93.9)	
mMRC symptom severity; n (%)				**< 0.001**
0–1	100 (39.4)	33 (73.3)	66 (32.4)	
≥2	154 (60.6)	12 (26.7)	138 (67.6)	
Number of exacerbations in the last 12 months; median (mix-max)	1 (0–4)	1 (0–2)	1 (0–4)	**0.033**
Number of hospitalisation in the last 12 months; median (min-max)	0 (0–3)	0	0 (0–3)	0.100
GOLD 2017 – CAT; n (%)				**0.003**
A	11 (5.4)	5 (14.3)	6 (3.6)	
B	115 (56.4)	21 (60.0)	90 (54.5)	
C	3 (1.5)	2 (5.7)	1 (0.6)	
D	75 (36.8)	7 (20.0)	68 (41.2)	
GOLD 2017 – mMRC; n (%)				**<0.001**
A	57 (27.5)	18 (50.0)	39 (23.1)	
B	71 (34.3)	9 (25.0)	60 (35.5)	
C	20 (9.7)	7 (19.4)	13 (7.7)	
D	59 (28.5)	2 (5.6)	57 (33.7)	
Type II Diabetes Mellitus; n (%)	28 (11.0)	3 (6.7)	25 (12.3)	0.279
Hypertension; n (%)	186 (72.9)	26 (59.1)	156 (76.5)	**0.018**
Hyperlipidaemia; n (%)	63 (24.6)	10 (22.2)	51 (25.0)	0.695
Coronary Heart Disease; n (%)	32 (12.5)	2 (4.4)	30 (14.7)	0.063
Thyroid disorder; n (%)	19 (7.4)	4 (8.9)	15 (7.4)	0.757
Osteoporosis; n (%)	7 (2.7)	1 (2.2)	5 (2.5)	0.999
Depression; n (%)	23 (9)	2 (4.4)	21 (10.3)	0.391
Gastro-oesophageal reflux; n (%)	30 (11.7)	9 (20.0)	21 (10.3)	0.07
Cancer (any); n (%)	3 (1.2)	1 (2.2)	2 (1.0)	0.452
≥2 comorbidities; n (%)	108 (42.5)	12 (27.3)	95 (46.8)	**0.028**

^a^*P*-value is given by Chi-square test or Fisher exact test for categorical variables and by T-test for normally distributed variables (age, BMI) or Mann Witney test for not normally distributed variables

^b^pooled group of 208 (82.2%) patients including: 15 (6%) frail subjects without disability, 162 (64%) frail with mobility disability and 31 (12.2%) subjects with mobility disability only. Disabled group as defined by FiND (A + B ≥ 1) consisted of the latter two groups (*n* = 193 (76.2%))

Bold faceted *P*-values show significant associations (P-value< 0.05). GOLD 2017: Global Initiative for Obstructive Lung Disease 2017 Guidelines, CAT: Chronic Obstructive Pulmonary Disease Assessment Test, mMRC: Modified Medical Research Council Dyspnoea Scale, BMI: body mass index

Two hundred and fifty three COPD patients had complete FiND data. In the analysis 45 (17.8%) robust subjects were compared to a pooled group of 208 (82.2%) patients including: 15 (6%) frail subjects without disability, 162 (64%) frail with mobility disability and 31 (12.2%) subjects with mobility disability only. Disabled group as defined by FiND (A + B ≥ 1) consisted of the latter two groups (*n* = 193 (76.2%)). Frailty was associated with age, hypertension, number of comorbidities, uncontrolled disease and number of exacerbations as well as with GOLD A-D status (Table 2). These associations remained significant when 3 groups were compared e.g., robust, frail, disabled (data not shown). Results of univariate logistic regression are presented in Table 3. Risk of frailty was significantly increased with age (OR (95%CI) 1.05 (1.02–1.08) per year), hypertension (OR (95%CI) 2.25 (1.14–4.45)), ≥ 2 comorbidities (OR (95%CI) 2.35 (1.14–4.81)), uncontrolled disease (OR (95%CI) for ≥10 CAT score 4.65 (1.86–11.63), or ≥ 2 mMRC score 5.75 (2.79–11.85), or ≥ 2 exacerbations 1.73 (1.07–2.78)), smoking cessation (ex-smokers vs. current smoking: OR (95%CI) 2.37 (1.10–5.28)) and GOLD B&D status (OR (95%CI) CAT-based: (4.65 (1.86–11.63); mMRC-based: 5.75 (2.79–11.85)). In a multivariate analysis (Table 4) only smoking cessation and GOLD status remained significant. To identify which component of GOLD stage (exacerbations or health status) was the main determinant of frailty we included in the multivariate regression CAT or mMRC score and exacerbations instead of CAT- or mMRC-based GOLD disease status. This sensitivity analyses showed that in the model including mMRC score and exacerbations the main significant determinant of frailty was mMRC (mMRC *P* = 0.001 and exacerbations *P* = 0.124). In the model including CAT score and exacerbations, both had a borderline significant association (*P* = 0.063). Multivariate analysis including ≥2 comorbidities instead of hypertension did not alter the results. Gender, BMI, occupation, symptom type or time and other comorbidities were not significant.

**Table 3:** Univariate analyses of frailty association with patient characteristics

	Crude OR (95%CI)
Age (years)	1.05 (1.02–1.08)
Marital status	
Married/with partner	ref.
Widow/Divorced/Never married	0.77 (0.33–1.81)
Occupational status	
Currently Employed	ref.
Retired/Unemployed/Housewife	2.04 (0.99–4.19)
Smoking status	
Current	ref.
Ex	**2.37 (1.07–5.28)**
Never	1.52 (0.54–4.29)
Hypertension	**2.25 (1.14–4.45)**
Coronary Heart Disease	3.71 (0.85–16.12)
Gastro-oesophageal reflux	0.46 (0.19–1.08)
≥2 comorbidities	**2.35 (1.14–4.81)**
CAT symptom severity	
<10	ref.
≥10	**4.65 (1.86–11.63)**
mMRC symptom severity	
0–1	ref.
≥2	**5.75 (2.79–11.85)**
Number of exacerbations in the last 12 months	
**< 2**	ref.
≥2	**1.73 (1.07–2.78)**
GOLD 2017 – CAT	
A&C	ref.
B&D	**5.64 (1.83–17.33)**
GOLD 2017 – mMRC	
A&C	ref.
B&D	**5.11 (2.34–11.16)**

GOLD 2017: Global Initiative for Obstructive Lung Disease 2017 Guidelines, CAT: Chronic Obstructive Pulmonary Disease Assessment Test, mMRC: Modified Medical Research Council Dyspnoea Scale. ref.: reference group Bold faceted OR (95%CI) shows significant associations (*P*-value< 0.05)

**Table 4:** Multivariate analysis of frailty association with patient characteristics

	GOLD 2017 CAT	GOLD 2017 mMRC
	adjusted OR (95%CI)	adjusted OR (95%CI)
Age (years)	1.03 (0.99–1.08)	1.04 (0.99–1.08)
Smoking status		
Current	ref.	ref.
Ex	**3.78 (1.19–12.02)**	**3.32 (1.03–10.71)**
Never	2.19 (0.44–10.86)	1.39 (0.34–5.67)
Hypertension	0.66 (0.27–1.65)	0.48 (0.19–1.22)
GOLD 2017 – CAT		
A&C	ref.	Na
B&D	**3.95 (1.09–14.34)**	
GOLD 2017 – mMRC		
A&C	na	ref.
B&D		**5.21 (2.19–12.44)**

na: not applicable, ref.: reference group, GOLD 2017: Global Initiative for Obstructive Lung Disease 2017 Guidelines, CAT: Chronic Obstructive Pulmonary Disease Assessment Test, mMRC: Modified Medical Research Council Dyspnoea Scale. Bold faceted OR (95%CI) shows significant associations (*P*-value< 0.05)

## Discussion

The objectives of the present study were to determine the prevalence of frailty in COPD patients visiting primary care facilities in Greece, and to identify the associated risk factors. Using the FiND questionnaire we found that frailty affects 82% of Greek COPD patients with a mean age of 67 years old, with the great majority of frail subjects suffering from mobility disability. The prevalence of frail-disabled COPD patients increased with age, GOLD stage or uncontrolled disease (CAT or mMRC or exacerbations), smoking cessation and comorbidities, but only GOLD stage and smoking cessation remained significant in multivariable analysis.

In out study the prevalence of frailty in COPD was greater compared to other populations, however this may be attributed to different definitions of frailty used and variation in population’s mean age, therefore it may be difficult to compare prevalence across studies. Several studies show that the prevalence of pre-frailty (defined as the presence of 1 or 2 modified Fried criteria [3]) is around 47% but reported prevalence of frailty in the general population and COPD enormously varied (range 4.0–74%) [6, 15, 18]. A study using survey data found that 57% of COPD patients suffered from frailty [8]. A general Dutch population of community-dwelling elderly able to perform the frailty tests, showed that 163 (5.8%) participants were frail and 1454 (51.3%) pre-frail [9]. In another cross-sectional study of COPD patients in Southeast Asia the occurrence of frailty and pre-frailty were 6.6 and 41.3% respectively [26]. In our study, the population was recruited in rural areas in Greece, where the living environment may contribute to the frailty profile, thus potentially affecting our findings. Nevertheless, the FiND questionnaire [20] used in our study includes 3 of the 5 original criteria (i.e. involuntary weight loss, low physical activity and exhaustion) of the frailty phenotype widely used making it very similar.

The FiND questionnaire is to support the identification of community-dwelling older persons presenting an increased risk of frailty in the early stage of the syndrome e.g., before developing mobility disability [20]. Unfortunately, we showed that the majority of COPD subjects in our study already had developed mobility disability, suggesting that screening of frailty in patients with COPD should start early and must be performed in multiple occasions to assess frailty status stage over time.

A prospective cohort study from respiratory outpatient and pulmonary rehabilitation clinics in the United Kingdom examined whether frailty affects completion and outcomes of pulmonary rehabilitation and found that frail patients had a two fold greater risk of programme non-completion. However, frailty could be reversed in the short-term in frail patients who managed to complete rehabilitation [27]. This promotes the idea of focusing more on pulmonary rehabilitation, and selecting rehabilitation approaches which may have components shared with treatments proposed for frailty management including exercise, nutritional support, self-management strategies and reduction of polypharmacy [4].

Α UK study showed that prevalence of frailty increased with age, GOLD stage, mMRC score and age-adjusted comorbidity burden [27]. Our study findings confirmed the aforementioned risk factors. Similarly Park et al. and Lahouse et al. showed that individuals with COPD who were older, had self-reported shortness of breath and comorbidities had increased risk of becoming frail, and frail people tended to have more disabilities [8, 9].

In COPD, shortness of breath and severity of symptoms may be linked to reduced physical activity, which may result in loss of muscle mass and strength, problems with mobility, therefore leading to frailty [27, 28].

We found that health status (CAT or mMRC scores) is a stronger predictor of frailty compared to the number of exacerbations. This may reflect the fact that frailty depends more on the long-term health status of COPD patients with its associated physical inactivity and muscle weakness, rather than on the short-term effect of change in physical activity due to an exacerbation. Identifying frailty early in the course of disease is important, as interventions can then be introduced to try to prevent further decline, hospital admission or death in those at high risk. Likewise, pulmonary rehabilitation should be introduced as early as possible in the course of COPD disease, for preventing or reversing frailty in COPD subjects.

Importantly, BMI did not differ significantly between frailty groups, which is similar to previous findings [9, 26, 29, 30], suggesting that ‘sarcopenic obesity’ might be a risk factor of frailty. ‘Sarcopenic obesity’ is the condition where lean body mass is lost, while fat mass may be preserved or even increased [31]. Thus muscle weakness may be attributed not only to loss of muscle tissue, but also to fat accumulation.

According to several reports, frailty increases with age [6]. Our study and a cross-sectional study in Thailand [26] showed that association with age did not remain significant in multivariate regression. This may suggest that it is the decline in respiratory function as we age that may increase the risk of frailty and not the age per se.

Similarly, hypertension was found to be a significant predictor of frailty in univariate logistic regression, but did not remain significant in adjusted models. We also observed a significant association between frailty and the number of comorbidities, which is consistent with other findings [3, 9, 26, 27]. A clear distinction between frailty and comorbidity has been described in the literature [1]. Frailty and comorbidity may interact and there can be synergistic effects on health-related outcomes, when frailty and comorbidity coexist.

A systematic review in 2015 including five cohort studies on smoking and frailty showed that baseline smoking significantly increased the risk for a worse frailty status at follow-up, even though most of the studies included had different methodology and frailty criteria used. The authors also reported that smoking cessation may potentially be beneficial for preventing or reversing frailty [32]. However, all of the studies compared never smokers with current or ex or ever smokers (current and ex smokers pooled). None of the studies compared frailty between ex and current smokers as we did. In our study never smoking was not associated with reduced risk of frailty compared to current smokers but ex-smokers were more likely to have frailty/disability than current smokers. We hypothesized that this may be due to the fact that our patients were extremely frail with an impaired health status and exacerbations, and they may be more likely to make the decision to quit smoking.

### Strengths and limitations of the study

This study enrolled patients from usual consultations in primary care independent of the disease severity, therefore findings may be applicable to a wider population. However, most of the primary care facilities in Greece until 2016 were based in semi-urban or rural areas which may limit the external validity of the study, and may not reflect the management from secondary-tertiary care. The FiND questionnaire, used in this study to screen frailty, is originally validated in patients above the age of 60 only, but in the current study we also included patients younger than 60 years old. Most of the existing tools that screen for frailty are validated in elderly or very elderly general populations and most of them are not designed to be self-administered [18, 19]. Although frailty has been associated with multimorbidity in the general population, studies exploring the issue of frailty in COPD are fewer, and some of those includes subjects younger than 60 years old [15]. However in the majority of observational studies the Fried criteria were used [15]. As we could not find any self-administered, community screening tool that had been validated in COPD or in middle age Caucasian adults, we decided to keep younger COPD subjects in the current analysis as in previous studies. However, FiND categorisation of COPD patients resulted in a very small group of frail subjects, therefore we had to pool those with the disabled patients group. Even though the majority of COPD subjects suffering with mobility disability were also frail based on FiND, our approach may limit the external validity of our study. Furthermore, the COPD diagnosis was based on patient records, therefore introducing risk of misclassification bias. Unfortunately, this study is cross-sectional in nature; thus, we were unable to explain the causal relationship between frailty and the identified risk factors.

Nevertheless, this study may have implications on clinical practice and public health as it the first study in Greece showing that frailty with mobility disability is common among COPD patients, and one of the first studies suggesting that severity of COPD disease increases the risk of frailty, while frail patients are more likely to quit smoking because of their disease severity.

### Implications of the study

The importance of assessing frailty in elderly patients and in those with multimorbidity has been included in several guidelines such as the National Institute for Health and Care Excellence (NICE) (for multimorbidity) [33] and joint actions on the management of other chronic diseases such as diabetes [34]. Frailty however is a neglected area of research and implementation in daily clinical practice in respiratory diseases. Given that the clinical impact of patients with frailty could be reversed with early interventions (especially at the pre-disable stage), early recognition of this syndrome among COPD patients is critical. Routinely, assessing frailty by a structured questionnaire in addition to the CAT, mMRC could provide additional important information to the doctors and allow early interventions, including physical and respiratory rehabilitation, referrals to geriatric and nutritional specialists, to take place. This could potentially prevent or delay the progression of frailty and improve COPD.

## Conclusions

Frailty with mobility disability is common in COPD patients and severity of disease increases the risk. Therefore routine assessment of frailty in addition to COPD control may allow for early interventions which may help delay progression of frailty and improve in COPD disease.

## Abbreviations

BMI: body mass index
CAT: COPD Assessment Test
COPD: chronic obstructive pulmonary disease
FiND: Frail non-Disabled questionnaire
GOLD: Global Initiative for Chronic Obstructive Lung Disease
mMRC: Modified Medical Research Council Dyspnea Scale
UNLOCK: Uncovering and Noting Long-term Outcomes in COPD and asthma to enhance Knowledge
